# Prognosis of hepatocellular carcinoma metastasizing to the oral cavity

**DOI:** 10.1186/s40902-021-00294-7

**Published:** 2021-03-10

**Authors:** Jun-Hee Hong, Keonmo Lee, Jinhong Kim, Kang-Min Ahn

**Affiliations:** Department of Oral and Maxillofacial Surgery, College of Medicine, University of Ulsan, Asan Medical Center, 88 Olympic-ro, 43-Gil, Songpa-gu, Seoul, 05505 South Korea

**Keywords:** Cancer, Hepatocellular carcinoma, Oral metastasis, Survival, Metastatic cancer

## Abstract

**Background:**

Oral metastasis by hepatocellular carcinoma (OMHCC) is extremely rare, and the prognosis had been reported quite poor due to simultaneous multiple organ metastases. In this study, we report clinical features and survival of 10 new cases of OMHCC and suggest the criteria for palliative surgery.

**Methods:**

A retrospective clinical study including 10 new cases of oral OMHCC between 2006 and 2016 was performed. Clinical features and survival analysis were examined. The recorded variables were age, sex, site of oral metastases, size of oral tumor (largest diameter), and survival after oral histopathologic diagnosis.

**Results:**

There was male (*n*=8) predilection of OMHCC. The mean survival time was 16.9 months. Patient age ranged from 40 to 71 years (mean 56.5). Eight mandibular and two maxillary lesions were found. One patient showed simultaneously the maxilla and the oral tongue involvement. The most often encountered symptoms were swelling (80%) followed by pain (60%), numbness (60%), bleeding (10%), and tooth mobility (10%). Four patients underwent operation due to spontaneous bleeding and swelling of the cancer. Overall (from onset of hepatocellular carcinoma) and truncated survival (from onset of OMHCC) were 71.9 and 13.1 months respectively.

**Conclusion:**

The prognosis of OMHCC was quite poor. Oral and jaw bone examination should be included in patients with multiple metastasis of HCC. Palliative surgery might be performed in patients who reported spontaneous bleeding, severe pain, and oral dysphasia due to tumor enlargement.

## Background

Hepatocellular carcinoma (HCC) is the sixth most common cancer [1] and the second leading cause of cancer mortality worldwide [2]. Incidence rates of HCC vary widely between geographic regions and are highest in Eastern Asia and sub-Saharan Africa [3]. HCC has a tendency to metastasize to other organs such as bone and soft tissues at the advanced stage. Oral metastatic cancer from distant organ cancer is relatively rare compared with oral squamous cell carcinoma, and it consists of approximately 1% of all oral cavity cancers [4–6]. Such metastases can occur to both bone and the oral soft tissues. Oral metastasis of hepatocellular carcinoma (OMHCC) is extremely rare, with only 61 cases reported in the literature [7, 8]. In English literature, 41 cases of OMHCC involving the jaw have been reported [9]. Oral lesions are usually one of multiple sites for HCC metastasis. When OMHCC is found, it means mostly the patient is at advanced stage of the HCC. Treatment for OMHCC is usually palliative chemoradiotherapy or surgery; however, no treatment is also suggested due to poor prognosis. Palliative surgery is quite difficult due to the bleeding tendency of HCC, and it should be performed when the benefit is superior to risks.

The purpose of this study was to report clinical features of ten new cases with OMHCC in a tertiary medical center during the period of 2006 to 2016 and evaluate survival periods and prognosis and to suggest the criteria for palliative surgery.

## Methods

A retrospective study based on histopathological and medical records from patients diagnosed with OMHCC was carried out. Patients’ medical records having all the information regarding the primary tumors, oral metastatic location, clinical presentation, and histological confirmation were included. OMHCC were diagnosed in total of ten patients (8 male, 2 female) at the department of oral and maxillofacial surgery of our hospital from March 2006 to November 2016. Ethical approval for this study was gained from the Institutional Review Board of our hospital (S2019-0054-0001). The study was conducted in accordance with the Helsinki Declaration, and the data was used in such a manner that subjects cannot be identified directly or indirectly.

The recorded variables were age, sex, site of oral metastases, size of oral tumor (largest diameter), and survival after oral histopathologic diagnosis. Also, through the medical record review, diagnosis date of primary HCC, diagnosis date of OMHCC, diagnosis date of other metastatic HCC, date of expire, and whether they had HBV (hepatitis B virus), HCV (hepatitis C virus), liver cirrhosis, history of alcohol drinking habit, hypertension, diabetes mellitus, tuberculosis were examined.

A truncated survival (from onset of OMHCC to death) and overall survival (from the day of HCC diagnosis to their death or at the end of the follow-up period) were calculated. A descriptive analysis of the data was performed, along with a survival analysis using Kaplan–Meier curves (log-rank test for comparison among curves) regarding about surgery, radiotherapy, and sites of metastasis. Statistical program (IBM SPSS ver. 22.0, Chicago, USA) was used to calculate the statistics. The significance level (*p*-value) established was 0.05.

## Results

### Clinical features

**Fig. 1:**
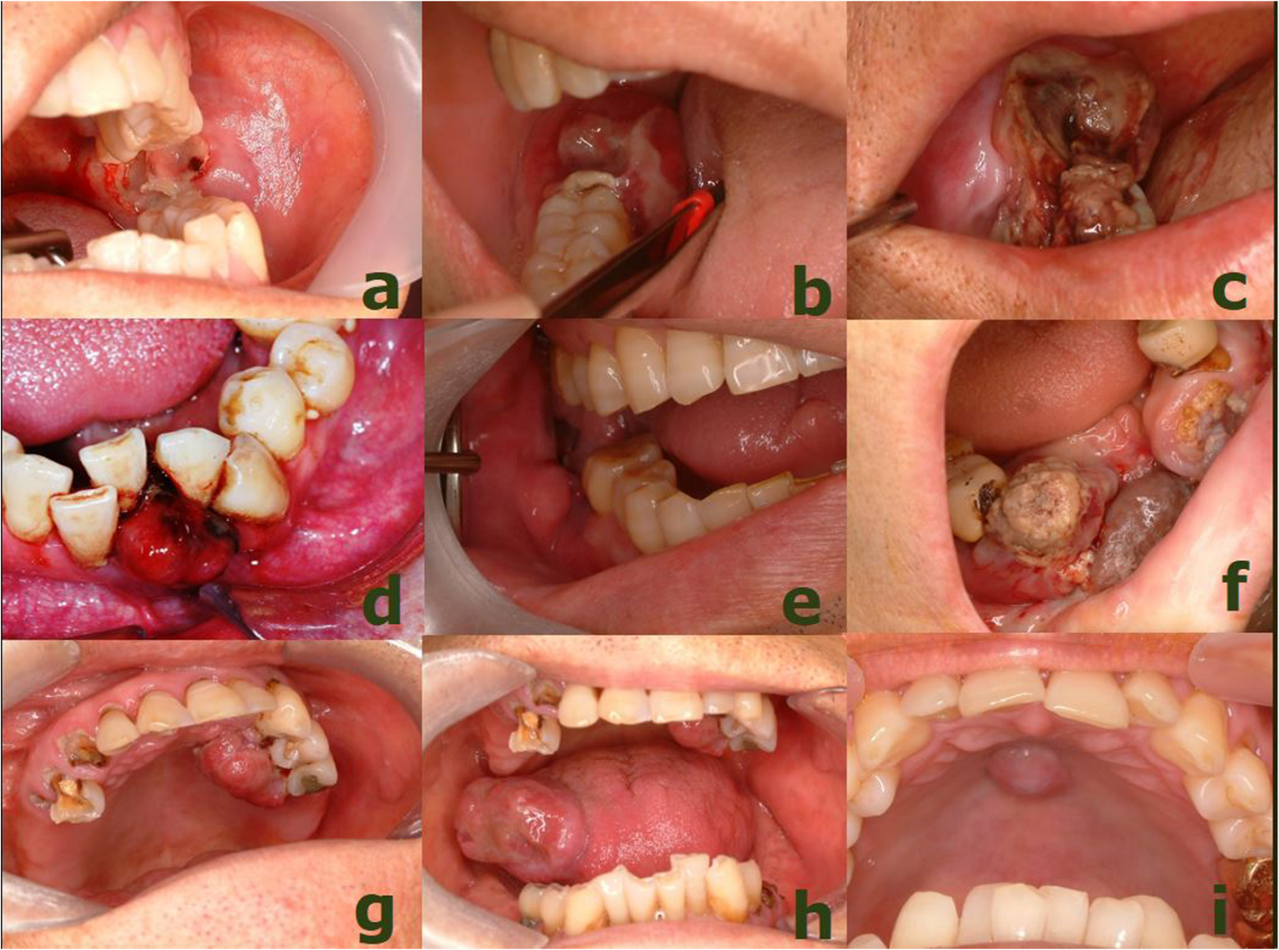
Oral metastasis by hepatocellular carcinoma. (**a** left mandible and retromolar trigone, **b**, **c** right mandibular gingiva, **d** anterior mandibular gingiva, **e** right mandibular gingiva, **f** anterior mandibular gingiva, **g** left maxillary palatal gingiva, **h** right lateral tongue, **i** midpalatal mucosa)

There were ten patients who were diagnosed with OMHCC (Table 1, Fig. 1). We found a male predilection (8 men and 2 women). The mean age was 56.5±11.1 years (range, 40 to 71 years). Eight mandibles and two maxillae were involved. One patient showed simultaneously the maxilla and the tongue metastasis. Four patients underwent operation to relieve their symptom, and the others were treated with palliative chemotherapy and/or radiotherapy. Among 10 patients, two patients visited in our clinic voluntarily, and the other eight patients were referred from their medical oncologist. All of the patients recognized their HCC. Nine patients already had other metastatic lesions other than oral cavity site, and one of them, the oral cavity was the first cancer metastatic site. One patient who had a single metastatic lesion in the oral cavity underwent operation.

**Table 1:** Summary of the survival and treatment of the 10 oral MHCC patients

No	A	S	Oral lesion	Other lesions	OOp (Y/N)	ORT (Y/N)	Chemo (Y/N)	SP(M)
	62	M	Mandible	Lung, bone	N	N	N	1
2	71	M	Mandible	Lung, brain	N	Y	Y	13
3	49	F	Mandible	Lung	N	N	Y	2
4	53	M	Mandible	Lung, bone	Y	Y	Y	12
5	40	F	Mandible	Bone	N	Y	Y	5
6	68	M	Maxilla	Lung	Y	N	Y	63
7	56	M	Mandible	Lung, brain, bone,	Y	N	Y	4
8	59	M	Mandible	Lung, adrenal gland, buttock	Y	Y	Y	40
9	40	M	Mandible	Abdomen	N	Y	Y	3
10	67	M	Mandible	Bone, adrenal gland	N	Y	Y	3

*A* Age, *S* Sex, *OOp* Oral operation, *ORT* Oral cavity radiotherapy, *Chemo* Chemotherapy, *SP(M)* Survival periods (months)

The most commonly encountered chief complaint was swelling (*n*=8) followed by pain (*n*=6), numbness (*n*=6), bleeding (*n*=1), and tooth mobility (*n*=1) (Table 2). All of OMHCCs were found to be the combined soft tissue-hard tissue lesion. There was no difference among right (*n*=4), left (*n*=3), and middle (*n*=3). In nine mandibular OMHCC, seven were posterior, and two were anterior lesion. In the maxillary lesion, it was located in the middle part of hard palate and palatal gingiva. The mean size of OMHCC was 3.3 ± 1.8 cm which measured in their largest diameter ranged from 0.8 to 7.2 cm. Oral lesion was the first manifestation of OMHCC in case 6 patient. In other patients, they already had some metastatic lesion other than oral site. The most often site other than oral site was the lung (*n*=7), followed by bone (*n*=4), brain (*n*=2), adrenal gland (*n*=2), buttock (*n*=1), and abdomen (*n*=1). There were nine patients who were found to be a HBV carrier. None was diagnosed with HCV infection. Eight patients had cirrhotic liver. Four patients had a heavy drinking habit before primary HCC diagnosis.

**Table 2:** Clinical details of the patients with oral metastasis by hepatocellular carcinoma

NO	Chief complaint	SL	TS	PS	RT	HCC survival	Meta survival	Others
1	Swelling, pain, numbness	Lt. Ramus (+Lt. RMT)	2.0	0	0	55	8	HBV, LC, drinking, HTN
2	Swelling	Lt. Ramus (+tongue)	4.0	0	50	90	15	HBV, DM
3	Numbness	Lt. mandibular body (+Lt. buccinator m.)	7.2	0	0	21	3	HBV, LC
4	Swelling, pain numbness	Rt. Ramus (+Rt. RMT)	3.5	1	40	25	14	HBV, LC, drinking
5	Pain, numbness	Rt. Ramus (+Rt. Masseter m.)	4.8	0	35	12	8	HBV
6	Swelling, pain	Midpalatal suture (+palatal mucosa)	0.8	5	0	233	43	HBV, LC, HTN, DM
7	Swelling, bleeding	Symphysis (+lower anterior gingiva)	2.0	2	0	21	15	HBV, LC, TBC
8	Swelling, pain, numbness, tooth mobility	Symphysis (+lower anterior buccal vestibule)	4.0	6	60	91	48	HBV,LC, drinking
9	Swelling, numbness	Rt. Ramus (+Rt. RMT)	2.5	0	35	13	4	HBV, LC
10	Swelling, pain	Rt. Ramus (+Rt. RMT)	2.0	0	40	16	16	LC, Drinking, HTN

*SL* Specific location of oral MHCC (+ involved soft tissue), *TS* Oral MHCC tumor size (cm), *PS* Times of palliative surgery, *RT* Total dose of palliative RT(Gy), *HCC survival* Survival period from Dx. Date of primary HCC (months), *Meta Survival* survival period from Dx. Date of other MHCC (months), *others* Other general condition, *Lt*. Left, *Rt*. Right, *RMT* Retromolar trigone, *HBV* Hepatitis B virus infection, *LC* Liver cirrhosis, *Drinking* History of having a heavy drinking habit, *HTN* Hypertension, *DM* Diabetes mellitus, *TBC* Tuberculosis

### Survival analysis

**Fig. 2:**
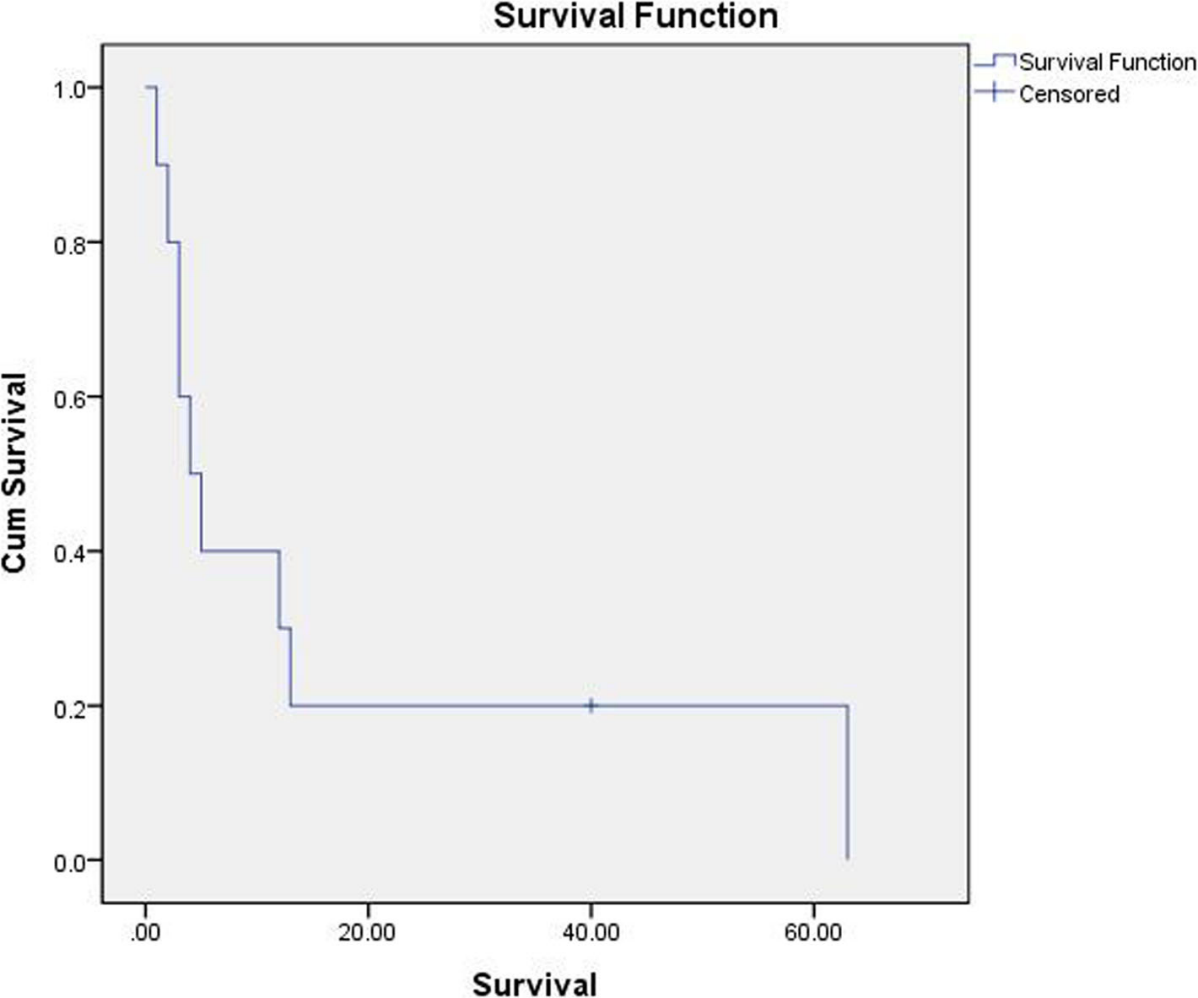
Mean survival time among patients with OMHCC

**Fig. 3:**
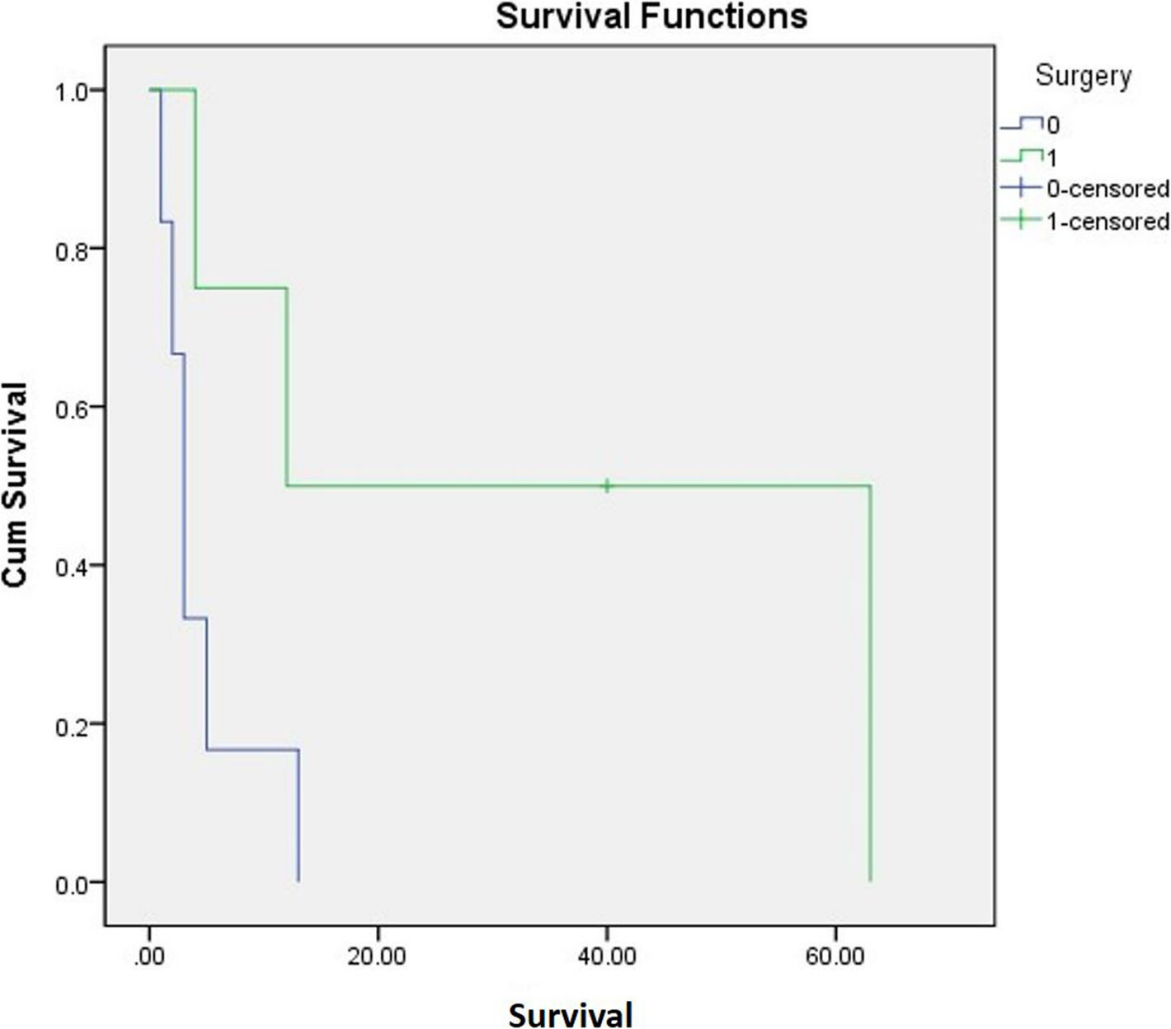
Mean survival time for other MHCC

**Fig. 4:**
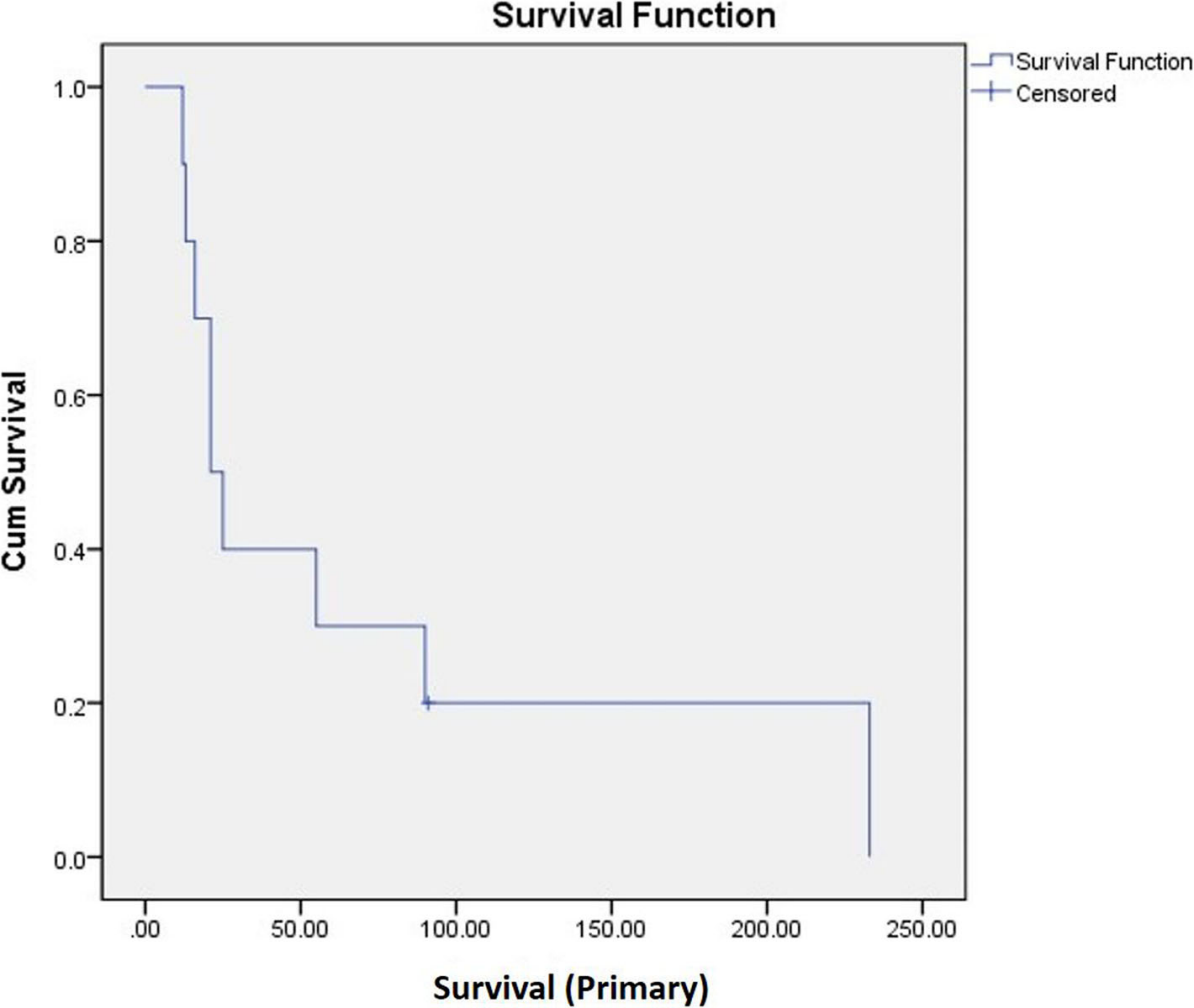
Mean survival time for primary HCC

Among ten OMHCC patients, only one patient was alive at the end of the study. Their mean survival time was 13.1±19.6 months (95% confidence interval (CI) = 1.5–32.2) (Fig. 2). Four patients who fulfilled the inclusion criteria of surgical approach underwent palliative operation. Six patients received radiotherapy on OMHCC with total dose ranged from 35 to 60 Gy (Table 2). There was no significant difference in survival between the group treated with oral radiotherapy or not (Log-rank 0.27; *p* > 0.05). One patient who reported the side effect of anti-cancer drug (azotemia) stopped palliative chemotherapy. The other nine patients received systematic chemotherapy for palliation. Six patients were treated with sorafenib, one patient sunitinib, one patient cisplatin, and one patient 5-FU. Among 10 patients, three patients underwent palliative surgery, and their mean survival time (35.5 months) was far longer than that of others (4.5 months). However, there was no significant difference between the mean survival times between two groups statistically (*p* = 0.059). The mean survival time for other MHCC was 17.4 months (95% CI = 8.2–26.6) (Fig. 3). When compared to that of OMHCC (13.1 months), the difference was not statistically significant (log rank 0.65; *p*>0.05). The mean survival time for primary HCC was 71.9 months for the 10 patients (Fig. 4). The average time between the diagnosis of the primary HCC and the diagnosis of the oral MHCC was 43.1 ± 50.5 months.

## Discussion

Patients suffering HCC with bone metastasis have a very poor survival rate; 1-year survival was 15–20%, and 2-year survival was only 4% [10]. Pesis et al. analyzed 41 patients from the English literature and reported that the average patient survival was 12.4 months after the diagnosis of primary HCC and 6.1 months after jaw metastasis [9]. In our study, we found a mean period of 71.9 months survival time after the diagnosis of primary HCC and a mean period of 16.9 months survival time after detection of OMHCC, which was a much better result than the previous report. There are two reasons to explain this difference. First, the time of the sample collection might have affected the survival. In the study of Pesis et al., patients were drawn from the literature published between 1940 and 2013; however, observation period of our patients is confined to the only last decade (2006~2016). The recent advancement of medical technology might have enabled early detection and intervention of involved region. Recent report by the National Cancer Institute in the USA showed that the 5-year relative survival rates for liver cancer were 5.6%, 8.5%, 11.2%, 15.3%, and 18.1% from 1993–1995, 1996–1998, 1999–2001, 2002–2005, and 2006–2012, respectively [11]. It reflected the progress in cancer therapy, and the data showed better results year by year. Second, the location of the sample collection would have influenced the survival. The epidemiology of HCC varies from country to country [12]. Especially, the relative contribution of HBV and HCV to HCC varies substantially country by country [13]. In this study, there were nine patients who could be classified as HBV-related HCC and one patient as alcoholic liver disease-related HCC. In South Korea, one study reported that the underlying liver diseases of HCC patients included HBV (72.3%), HCV (11.6%), alcoholic liver disease in (10.4%), and non-B non-C hepatitis (0.7%) [14–16]. Our case is consistent with this report. In the USA, another study reveals that the underlying liver diseases of HCC patients included hepatitis B (23%), hepatitis C (39%), alcoholic liver disease in (21%), and non-B non-C hepatitis (12%) [17] Diagnostic and management methods such as medication, vaccination, and detection of cirrhotic liver are influenced by these hepatitis types [18]. Early detection of tumor through proper diagnosis and surveillance is critical to patient survival. Variation in management including treatment patterns throughout the world and whether the nation had government-funded or national surveillance programs would be also sequential to patients’ survival.

In spite of the longer survival time than previously reported, 16.9 months is not enough time for the patients diagnosed with OMHCC. A poor prognosis should be noticed to the patients and their family. Among 10 patients, four underwent palliative surgery to control the oral lesion and others not. Patients who underwent palliative oral surgery lived more (35.5 months) than the others who did not received operation (4.5 months). Although there was no statistically significant difference (*p* = 0.059) in mean survival time between the two groups due to the limited sample size (*n*=10), huge difference in survival time (31.0 months) existed. That might have come from the decision-making process. When we decide to do the surgery or not, the main reason for not performing the surgery was that the surgery would not help the patient’s survival. Collaboration with oncologist is always recommended because the life expectancy of OMHCC patients is often very short.

Before deciding to perform the palliative surgery or not, the risk of surgery should be evaluated by appropriate clinical examination. Palliative surgery should be performed when the benefit is way superior to the risk. The decision for operation should be made based on the clinical examination of patients’ both oncologist and oral and maxillofacial surgeon. The main reason and inclusion criteria for operation are (1) spontaneous bleeding which might jeopardize the patient unless treated, (2) movable mass which makes it difficult to chew and swallow, (3) severe pain, and (4) single metastasis from HCC. When it comes to OMHCC, the risk of massive bleeding is always present. Metastasis from HCC has been reported as a hemorrhagic tumor because of its hypervascular nature [19]. Furthermore, in view of the coagulopathy that often accompanies primary liver disease, a resection or even a biopsy can be complicated by hemorrhage. In our cases, there was no severe coagulopathy. The INR (Internationalized Normalized Ratio) of all patients ranged from 0.94 to 1.21. However, all four patients undergoing surgery had a large amount of bleeding. Meticulous bleeding control was always required.

In our cases, there was just 1 case that oral lesion was the first manifestation of MHCC. In other 9 patients, they already had some metastatic lesion other than oral site. This means that proper management on oral lesion could not increase patients’ survival rate dramatically in 9 cases. However, in case 6 which was oral lesion as first manifestation of metastasis from HCC shows longest survival times (63 months). It seemed that proper management of oral lesion was beneficial to the patient survival time. In a previous review including 390 metastatic tumors to the jaw bones, the average time between the diagnosis of the primary tumor and appearance of the jawbone metastases was estimated to be 39.5 months [20]. It is similar as our cases which show that the average time between the diagnosis of the primary HCC and the diagnosis of the OMHCC was 43.1 ± 50.5 months. Among 10 patients, 6 patients received radiotherapy on OMHCC with total dose ranged from 35 to 60 Gy. Radiotherapy is generally considered effective for metastatic bone cancer, and there are reports concerning the effects of radiotherapy on bone metastases from HCC [21]. In a recent study of radiotherapy on metastasis from HCC, total dose ranged from 12.5 to 66 Gy resulted in pain relief rate from 73 to 99.5% and improvement of the patient’s quality of life [22]. The 1-year survival rate after radiotherapy initiation or the diagnosis of bone metastasis of HCC has been reported to be 13.8–32.4%, with a 5–7.4 months’ median survival time [7, 8, 23]. In our cases, the median survival time of patients treated with radiotherapy for oral lesion was 5 months. It is same as the result above. However, in our cases, due to the limited sample number (*n*=10) and multifactorial effect on patient survival, there was no significant difference between the group treated with oral radiotherapy or not (Log-rank 0.27; *p* > 0.05).

Except one patient due to the side effect of anti-cancer drug (azotemia), 9 patients were on systematic chemotherapy (6 patients treated with sorafenib, 1 patient sunitinib, 1 patient cisplatin, 1 patient 5-fluorouracil). Again, in our cases, due to the limited sample number (*n*=10) and multifactorial effect on patient survival, it is difficult to evaluate the relationship between kind of systemic chemotherapy and patient survival in OMHCC cases. There was a certain male predilection (8 men and 2 women), and this corresponds with the male predominance of HCC. Rates of liver cancer among men are two to four times as high as the rates among women [2]. Patient age ranged from 40 to 71 years (mean 56.5, median 57.5) in this study. In case of HCC, the greatest proportional increase has been seen among Hispanics and Whites between 45 and 60 years of age [24]. It is also similar to the mean age of all head and neck metastases [25]. The most often encountered chief complaint was swelling (80%) followed by pain (60%), numbness (60%), bleeding (10%), and tooth mobility (10%). This corresponds with the recent study of metastasis from HCC to the jaw bone [9]. Metastasis from HCC to the jaw is very rare. When it does occur, the most frequently affected site is the mandible [26]. Among 10 OMHCC cases, the mandibular were affected in 9 cases (90%), and there was a significant predilection over maxilla (10%). In 9 mandibular OMHCCs, seven were posterior and two were anterior lesion. However, in the maxillary lesion, it was located in the middle part of hard palate anterior-posteriorly. The mean size of OMHCC was 3.3 ± 1.8 cm which measured in their largest diameter ranged from 0.8 to 7.2 cm. This corresponds with the recent study about OMHCC to the jaw bone reporting that the size of lesion ranged from 0.2 to 9 cm (mean 3.95) [9].

Generally, in metastatic tumors to the jaw bone, the mandible was the most common location (82%) with the molar area being the most frequently involved site [27]. This is because the mandible contains hematopoietic tissue especially in the posterior mandible [19]. Several pathways of HCC metastasis to the jaw have been suggested. Among them, hematogenous pathway is widely accepted; the tumor reaches the circulation through invasion of the hepatic arterial and/or portal venous branches. Most jaw metastases are associated with lung metastases, and they possibly occur by this route [28]. In our cases of OMHCC, the most often site other than oral site was the lung (7), followed by bone (4), adrenal gland (2), brain (2), buttock (1), and abdomen (1). In a recent study including 398 HCC autopsies, extrahepatic metastasis was found in 156 of 398 HCC (39.1%), with lung metastasis (74.5%) being the most common, followed by the bones (24.8%) and adrenal glands (19.1%) [29]. The proposed pathway of metastasis above corresponds to these results.

The Batson venous plexus is a network of valveless veins that connect the deep pelvic veins and thoracic veins to the internal vertebral venous plexuses. Metastatic HCC cells can reach the mandible through the Batson plexus bypassing the pulmonary, inferior caval and portal venous circulations. This pathway may be responsible for metastasis to the vertebral bodies, which are the preferred site of bony HCC metastasis. This could be the most likely pathway from HCC without pulmonary metastasis as appeared in 3 of 10 cases in this study. There were some cases that showed the relationship with oral surgery and tumor growth stimulation. In our patients, cases 6 and 8 who underwent many oral palliative surgeries suffered from tumor recurrence in surgical margin. Also, MHCC in case 9 emerged from surgical extraction of the right mandibular third molar.

## Conclusion

The prognosis of OMHCC was quite poor. Oral and jaw bone examination should be included in patients with multiple metastasis of HCC. Palliative surgery might be performed in patients who reported spontaneous bleeding, severe pain, and oral dysphasia due to tumor enlargement. When we determine to perform the operation, the risks of surgery like massive bleeding and impaired wound healing should be evaluated by appropriate clinical examination.

## Data Availability

All the data were retrieved from electronic medical chart of Asan medical center.

## Abbreviations

HCC: Hepatocellular carcinoma
OMHCC: Oral metastasis of hepatocellular carcinoma
HBV: Hepatitis B virus
HCV: Hepatitis C virus

## References

[1] Ferenci P, Fried M, Labrecque D, Bruix J, Sherman M, Omata M (2010). World Gastroenterology Organisation Guideline. Hepatocellular carcinoma (HCC): a global perspective. J Gastrointestin Liver Dis.

[2] El-Serag HB (2011). Hepatocellular carcinoma. N Engl J Med.

[3] Lange N, Dufour JF (2019). Changing epidemiology of HCC: how to screen and identify patients at risk?. Digestive Dis Sci.

[4] Hirshberg A, Buchner A (1995). Metastatic tumours to the oral region, An overview. Eur J Cancer B Oral Oncol.

[5] Jeon YT, Kim CH, Park SM, Kim MK (2019). Distant metastasis of follicular thyroid carcinoma to the mandible: a rare case report. J Korean Assoc Oral Maxillofac Surg.

[6] Kim IK, Lee DH, Cho HY, Seo JH, Park SH, Kim JM (2016). Prostate adenocarcinoma mandibular metastasis associated with numb chin syndrome: a case report. J Korean Assoc Oral Maxillofac Surg.

[7] Pires FR, Sagarra R, Correa ME, Pereira CM, Vargas PA, Lopes MA (2004). Oral metastasis of a hepatocellular carcinoma. Oral Surg Oral Med Oral Pathol Oral Radiol Endod.

[8] Doval DC, Kannan V, Kumaraswamy SV, Reddy BK, Bapsy PP, Rao CR (1992). Mandibular metastasis in hepatocellular carcinoma. Int J Oral Maxillofac Surg.

[9] Pesis M, Taicher S, Greenberg G, Hirshberg A (2014). Metastasis to the jaws as a first manifestation of hepatocellular carcinoma: report of a case and analysis of 41 cases. J Craniomaxillofac Surg.

[10] Kaizu T, Karasawa K, Tanaka Y, Matuda T, Kurosaki H, Tanaka S (1998). Radiotherapy for osseous metastases from hepatocellular carcinoma: a retrospective study of 57 patients. Am J Gastroenterol.

[11] Vitale A, Burra P, Frigo AC, Trevisani F, Farinati F, Spolverato G (2015). Survival benefit of liver resection for patients with hepatocellular carcinoma across different Barcelona Clinic Liver Cancer stages: a multicentre study. J Hepatol.

[12] Tang A, Hallouch O, Chernyak V, Kamaya A, Sirlin CB (2017) Epidemiology of hepatocellular carcinoma: target population for surveillance and diagnosis. Abdom Radiol (New York) 10.1007/s00261-017-1209-1 28647765

[13] de Martel C, Maucort-Boulch D, Plummer M, Franceschi S (2015). World-wide relative contribution of hepatitis B and C viruses in hepatocellular carcinoma. Hepatology (Baltimore, Md).

[14] Kim BH, Park JW (2018). Epidemiology of liver cancer in South Korea. Clin Mol Hepatol.

[15] Nam JY, Jang ES, Kim YS, Lee YJ, Kim IH, Cho SB (2020). Epidemiological and clinical characteristics of hepatitis C virus infection in South Korea from 2007 to 2017: a prospective multicenter cohort study. Gut Liver.

[16] Yim SY, Kim JH (2019). The epidemiology of hepatitis B virus infection in Korea. Korean J Intern Med.

[17] Park JW, Chen M, Colombo M, Roberts LR, Schwartz M, Chen PJ (2015). Global patterns of hepatocellular carcinoma management from diagnosis to death: the BRIDGE Study. Liver international : official journal of the International Association for the Study of the Liver.

[18] Singal AG, El-Serag HB (2015). Hepatocellular carcinoma from epidemiology to prevention: translating knowledge into practice. Clin Gastroenterol Hepatol.

[19] Ashar A, Khateery SM, Kovacs A (1997). Mandibular metastatic hepatocellular carcinoma: a case involving severe postbiopsy hemorrhage. J Oral Maxillofac Surg.

[20] Hirshberg A, Leibovich P, Buchner A (1994). Metastatic tumors to the jawbones: analysis of 390 cases. J Oral Pathol Med.

[21] Roca EL, Okazaki N, Okada S, Nose H, Aoki K, Akine Y (1992). Radiotherapy for bone metastases of hepatocellular carcinoma. Jpn J Clin Oncol.

[22] Hayashi S, Tanaka H, Hoshi H (2014). Palliative external-beam radiotherapy for bone metastases from hepatocellular carcinoma. World J Hepatol.

[23] Seong J, Koom WS, Park HC (2005). Radiotherapy for painful bone metastases from hepatocellular carcinoma. Liver Int.

[24] El-Serag HB, Lau M, Eschbach K, Davila J, Goodwin J (2007). Epidemiology of hepatocellular carcinoma in Hispanics in the United States. Arch Intern Med.

[25] Thiele OC, Freier K, Bacon C, Flechtenmacher C, Scherfler S, Seeberger R (2011). Craniofacial metastases: a 20-year survey. J Craniomaxillofac Surg.

[26] Teshigawara K, Kakizaki S, Sohara N, Hashida T, Tomizawa Y, Sato K (2006). Solitary mandibular metastasis as an initial manifestation of hepatocellular carcinoma. Acta Med Okayama.

[27] Hirshberg A, Shnaiderman-Shapiro A, Kaplan I, Berger R (2008). Metastatic tumours to the oral cavity - pathogenesis and analysis of 673 cases. Oral Oncol.

[28] Takinami S, Yahata H, Kanoshima A, Yamasaki M, Funaoka K, Nakamura E (1995). Hepatocellular carcinoma metastatic to the mandible. Oral Surg Oral Med Oral Pathol Oral Radiol Endod.

[29] Schlageter M, Quagliata L, Matter M, Perrina V, Tornillo L, Terracciano L (2016). Clinicopathological features and metastatic pattern of hepatocellular carcinoma: an autopsy study of 398 patients. Pathobiology.

